# Overexpression of MTHFD2 represents an inflamed tumor microenvironment and precisely predicts the molecular subtype and immunotherapy response of bladder cancer

**DOI:** 10.3389/fimmu.2023.1326509

**Published:** 2023-12-07

**Authors:** Xiaokai Shi, Xiangrong Peng, Yin Chen, Zebin Shi, Chuang Yue, Li Zuo, Lifeng Zhang, Shenglin Gao

**Affiliations:** ^1^ Department of Urology, ChangZhou No.2 People’s Hospital, Nanjing Medical University, ChangZhou, Jiangsu, China; ^2^ Laboratory of Urology, ChangZhou Medical Center, Nanjing Medical University, ChangZhou, Jiangsu, China

**Keywords:** bladder cancer, MTHFD2, inflamed tumor microenvironment, immunotherapy, molecular subtype

## Abstract

**Introduction:**

Methylenetetrahydrofolate dehydrogenase 2 (MTHFD2), whose aberrant expression is common in cancers, has recently been identified as a potential regulator of immune response. However, its immune-related role in bladder cancer (BLCA) and its association with immunotherapy efficacy remain unclear.

**Methods:**

RNA sequencing data from The Cancer Genome Atlas (TCGA) was applied to analyze the immunological roles and prognostic value of MTHFD2 in pan-cancers. The association of MTHFD2 with several immunological features of tumor microenvironment (TME), including cancer-immunity cycle, immune cells infiltration, immune checkpoints expression, and T cell inflamed score was analyzed in TCGA-BLCA cohort. The predictors of cancer treatments effectiveness, including the expression and mutation of certain genes, molecular subtypes, and several signatures were evaluated as well. These results were validated by another independent cohort (GSE48075). Finally, the predictive value of MTHFD2 for TME and immunotherapy efficacy were validated using immunohistochemistry assay and RNA sequencing data from IMvigor210 cohort, respectively.

**Results:**

MTHFD2 was found to be positively associated with several immunological features of an inflamed tumor microenvironment (TME) in various cancers and could predict BLCA patients’ prognosis. In BLCA, high expression of MTHFD2 was observed to be positively related with the cancer–immunity cycle, the infiltration of several immune cells, and the expression of immunoregulators and T-cell inflamed scores, indicating a positive correlation with the inflamed TME. Moreover, patients with high MTHFD2 expression were more likely to be basal-like subtypes and respond to BLCA treatments, including immunotherapy, chemotherapy, and target therapy. The clinical data of the IMvigor210 cohort confirmed the higher response rates and better survival benefits of immunotherapy in high-MTHFD2-expression patients.

**Conclusion:**

Collectively, high MTHFD2 predicts an inflamed TME, a basal-like subtype, and a better response to various therapeutic strategies, especially the ICB therapy, in bladder cancer.

## Introduction

Bladder cancer (BLCA) is the 10th and 6th most prevalent malignancy in male and female patients, respectively, causing more than 570,000 new cases and 210,000 deaths worldwide every year (1). Owing to the need for lifelong surveillance and invasive treatments, BLCA imposes immense suffering and economic burden on patients and the society (2). BLCA originates from the transitional epithelium and can be classified into two groups according to invasion depth: non-muscle-invasive BLCA (NMIBC) and muscle-invasive BLCA (MIBC) (3). While the prognosis of NMIBC patients has been favorable thanks to treatment advancement, patients with MIBC still suffer from a dismal prognosis (3). The 5-year survival of regional and distant metastatic MIBC patients is 36% and 5%, respectively (3).

Recently, immunotherapies, particularly the immune checkpoint blockade (ICB) therapy and chimeric antigen receptor T cell (CAR-T) therapy, have shown great success in several cancers and have shown promising survival benefits for advanced BLCA (4–10). However, these treatments only work for a small portion of patients (9, 10). Currently, there are several biomarkers that can predict the responsiveness of immunotherapy, such as the mRNA level of Programmed cell death 1 ligand 1(PD-L1), tumor mutation burden (TMB), microsatellite instability (MSI), and molecular subtypes (11–14). Nevertheless, all of these biomarkers have their own drawbacks, which greatly limit their use in clinical practice. For instance, the predictive accuracy of PD-L1 expression can be disturbed by lots of factors, and the detection of TMB, MSI, and molecular subtypes is complex, tardy, and expensive (11–15). Therefore, explorations for new predictive biomarkers remain urgent.

Methylenetetrahydrofolate dehydrogenase 2 (MTHFD2) is a vital enzyme involved in mitochondrial folate one-carbon metabolism and exerts a dual role of dehydrogenase and cyclohydrolase (16). It can catalyze the conversion of 5,10‐methylenetetrahydrofolate (CH2‐THF) and NAD(P)^+^ to 10‐formyl‐THF (CHO‐THF) and NAD(P)H, and thus participates in several biological processes, including the production of energy and the metabolism of nucleotide and amino acid (17, 18). Normally, MTHFD2 is heavily expressed in fetuses, but almost absent in adults (19). However, it can be upregulated in various cancers to meet the high biosynthetic requirements of rapid cell proliferation (19). Conversely, the absence of MTHFD2 in tumors may impair the malignant features of cancers and trigger cell death (19). The high expression of MTHFD2 is also correlated with the shorter survival of several cancers, including breast cancer, colorectal cancer, kidney cancer, and liver cancer (20–23), thus becoming a promising prognostic biomarker and therapeutic target (24).

However, recent studies have indicated that, except for metabolic functions, MTHFD2 is also involved in the regulation of the immune system in several diseases. The study of Sugiura et al. (25). demonstrated that MTHFD2 is vital to functions of T cells. While high MTHFD2 expression can support *de novo* purine synthesis of activated CD4^+^ T cells, insufficient MTHFD2 will promote Treg-like phenotypes of Th17 cells. Therefore, targeting MTHFD2 to decline its expression may be an ideal way to protect against inflammation and autoimmunity (25). In addition, another study has shown that MTHFD2 can upregulate the expression of PD-L1, thereby causing cancer immune evasion (26). These results revealed the possible association of MTHFD2 with cancer immunity and its potential to be a novel biomarker in cancer treatment. However, its detailed role in specific cancers including BLCA and its relationship with immunotherapy response remain obscure.

In this study, we performed a multi-omics analysis of immune-related functions of MTHFD2 in BLCA. We observed that MTHFD2 expression level is associated with the tumor microenvironment (TME) in BLCA and has the ability to precisely predict the molecular subtypes, inflamed TME, and immunotherapy response in bladder urothelial carcinoma.

## Methods

### Patients and tissue samples

Tumor tissue microarrays of BLCA (HBlaU050CS01), which added the staining results of CD8 and PD-L1, were purchased from Shanghai Outdo Biotech Company (Shanghai, China). CD8 is an antigen of cytotoxic T lymphocyte. The proportion of CD8^+^T cells’ positive rate was calculated as follows: number of CD8^+^ cells in the nest/number of all cells in the nest. Only the proportion of cells with a strong positive rate was recorded, and the proportion of cells with a weak positive rate was ignored. The study was approved by the Ethics Committee of Shanghai Outdo Biotech Company. The immunohistochemistry of the tumor tissue microarray was performed by Biossci Company, Hubei, China. The anti-MTHFD2 antibody (12270-1-AP) was purchased from Proteintech Company. Subsequently, two independent pathologists were asked to semiquantitatively score the intensity of immunohistochemical staining of MTHFD2 as negative (0), weakly positive (1^+^), moderately positive (2^+^), or strongly positive (3^+^) along with the percentage of positive cells. For each observed tissue component (the cytoplasm and nucleus), a summary value referred to as the component H-score was calculated by multiplying the intensity score (ranging from 0 to 3) and the percentage of positive cells (ranging from 0 to 100).

### Data collection and preprocessing

The transcriptional data (FPKM value), prognosis information, and genetic alteration data of pan-cancers were acquired from the UCSC Xena (https://xenabrowser.net/datapages/) database (27). Then, the FPKM data were transformed into TPM data with the following formula:


$$TPM=\frac{FPKM_{i}}{\Sigma_{j}FPKM_{j}}\times 10^{6}$$

Then, the log2^(TPM+1)^ transformation was further performed on the TPM value for subsequent analysis. The somatic mutation data were processed by VarScan (28) (https://varscan.sourceforge.net/) and calculated to TMB. Furthermore, we downloaded the MSI data from the cBioPortal (29) (http://www.cbioportal.org/) database. The copy number variation (CNV) data of hyperprogression marker genes were obtained from a previous study (30). Furthermore, three independent cohorts, namely, GSE48075, E-MTAB-4321, and IMvigor210 (http://research-pub.gene.com/IMvigor210CoreBiologies/), were downloaded for external validation (31–33). These data were also transformed into the TPM value for subsequent analysis.

### Assessment of immunological characteristics

The association between MTHFD2 expression and several immunological characteristics was analyzed. The gene lists of immune checkpoints and various immunoregulators and various immunoregulators, including chemokines, chemokine receptors, and major histocompatibility complex (MHC), were acquired from previous studies (34, 35). The cancer–immunity cycle, which reflects the stepwise events of immune systems’ response to cancer (36), was analyzed by single sample gene set enrichment analysis (ssGSEA) and normalized to *z*-score. The infiltration of TIICs was evaluated by ssGSEA in pan-cancer, and six other algorithms in BLCA, namely, TIMER (37), MCP-counter (38), CIBERSORT (39), quanTIseq (40), xCell (41), and EPIC (42), to enhance the credibility of results. While the ssGSEA was performed by the R package “GSVA” based on signatures from the TISIDB database (43), the results of the other six algorithms were obtained from the TIMER (http://timer.cistrome.org/) website. The expression of effector genes of these immune cells, which was obtained from a previous study (30), was assessed as well. Moreover, the T cell-inflamed score (TIS), a previously developed predictor of cancer immunity and efficacy of anti PD-1 therapy (44), was utilized to evaluate the degree of inflammation of the TME in BLCA as well. The TIS, which could reflect the pre-existing anticancer immunity and predict the clinical response of ICB, was calculated based on 18 IFN-γ responsive genes. These genes was collected from the research of Ayers et al.


$$TIS=\Sigma_{\gamma =1}^{18}\beta_{\gamma }X_{\gamma }$$

where β_γ_ is a weighted coefficient predefined in a previous study, and Xγ is the γth gene’s expression level. Moreover, the association between MTHFD2 and several previously reported hyperprogression predictive genes, whose aberrant expression or copy number variance can predict the occurrence of hyperprogression, was analyzed as well.

### Evaluation of the associations between MTHFD2 and various therapeutic efficacy

To determine the relationships between MTHFD2 expression and the efficacy of different therapies, we performed analyses on several predictive biomarkers. The expression or genetic alteration conditions of various previously reported predictive genes were evaluated, including genes for hyperprogression after immunotherapy (45–47), predictors for neoadjuvant chemotherapy responsiveness (48–51), and drug-targeted genes of different treatment strategies. The drug-targeted genes were downloaded from the Drugbank (52) database (https://www.drugbank.com/). Furthermore, a lot of previously established gene signatures with predictive power were examined by the ssGSEA algorithm as well (14, 33, 53–56). The GSVA package was utilized to assess the enrichment score of different signatures in various samples (57).

### Molecular subtype assessment

The molecular subtype of BLCA samples was determined by different subtyping systems, including UNC (58), Baylor (59), TCGA (60), MDA, Lund (61), CIT-Curie (62), and Consensus subtypes (14). The analyses were performed by the R package “BLCAsubtyping” and “ConsensusMIBC”.

### Statistics

Wilcoxon signed rank test or Kruskal–Wallis test was applied for the differences among continuous variables according to the number of groups, and the chi-square test was applied for the differences among classified variables. The correlation analyses were based on Pearson’s coefficients. The survival of patients was compared by both univariate Cox regression and the Kaplan–Meier method. All analyses were performed by R (version 4.2.3) and results with a two-sided *p*-value of less than 0.05 were considered significant.

## Results

### Pan-cancer analysis of MTHFD2 prognostic value and immunological association

**Figure 1:**
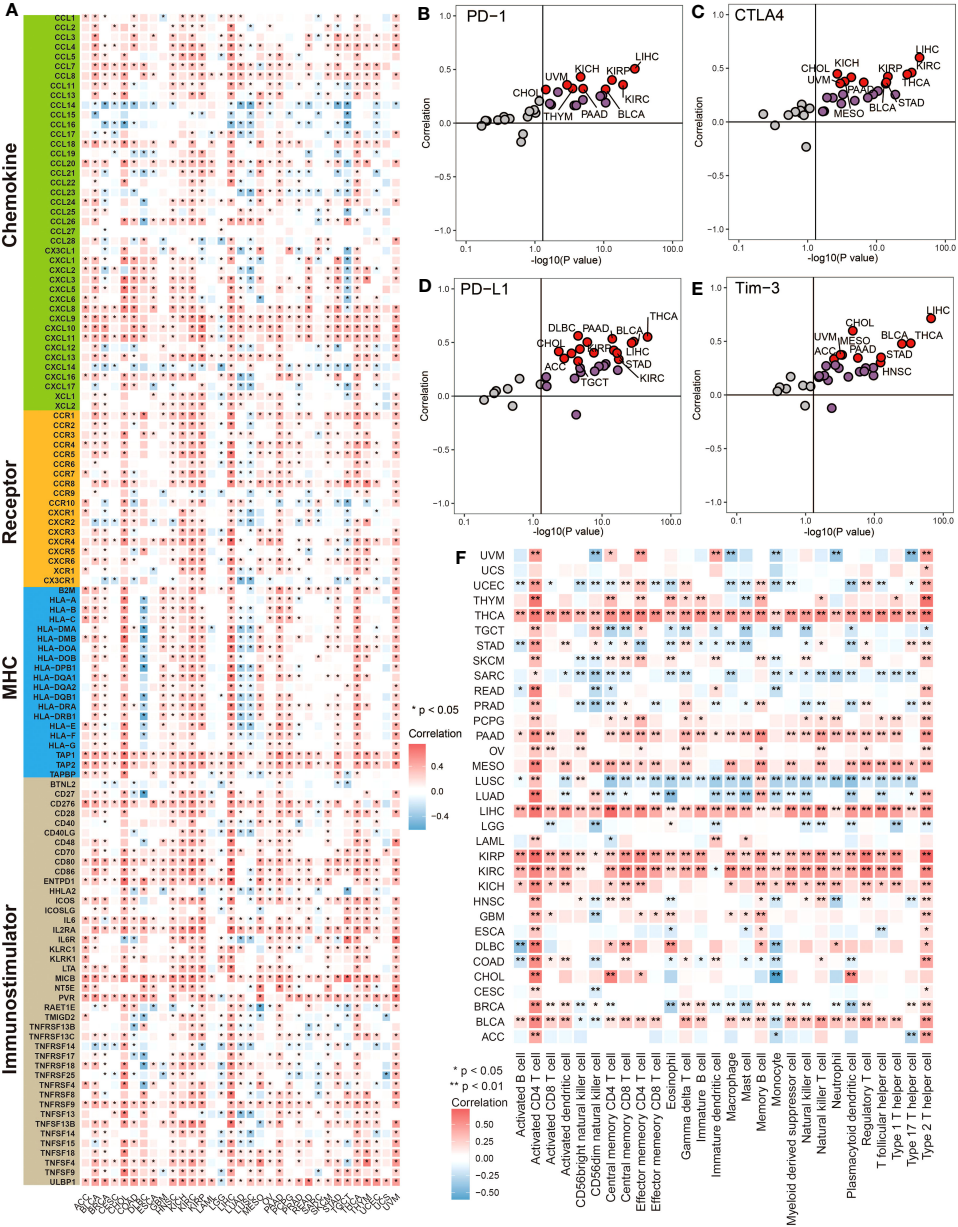
Pan-cancer analysis revealed the correlation between MTHFD2 and immunological status. **(A)** The heatmap for correlation between MTHFD2 and various immunomodulators in pan-cancers. Grids in red represent positive correlation while grids in blue represent negative correlation. (*p< 0.05). **(B–E)** The scatter plot for correlation between MTHFD2 and various immune checkpoints (PD-1, CTLA4, PD-L1, and Tim-3). Dots in red represent the absolute correlation > 0.3. **(F)** The heatmap for association between MTHFD2 expression and infiltration level of various immune cells in pan-cancers. Grids in red represent positive correlation while grids in blue indicate adverse correlation (*p< 0.05, **p< 0.01).

Pan-cancer analysis was performed to have a basic understanding of the biological functions and prognostic value of MTHFD2. Firstly, univariate Cox regression and survival curve was used to assess the prognostic value of MTHFD2 in terms of overall survival (OS), progression-free survival (PFS), and disease-free survival (DFS). As shown in **Supplementary Figure 1**, MTHFD2 overexpression had a significant risky role for OS in ACC, BLCA, BRCA, HNSC, KICH, KIRC, KIRP, LUAD, MESO, PAAD, PRAD, and UCEC via univariate Cox regression, while the protective role was only found in GBM, LGG, and SKCM. Similar results were observed in PFS analysis, except BRCA, GBM, MESO, and SKCM were no longer significant, while PCPG and UVM reached significance (**Supplementary Figure 2**). However, in DFS analysis, only KIRP, KICH, and PAAD reached significance (**Supplementary Figure 3**). Subsequently, we further investigated the correlation between MTHFD2 expression and immunological features. The expression of MTHFD2 was found to be positively correlated with numerous immunoregulators in most cancers, especially in BLCA, CHOL, KIRC, KIRP, LIHC, and THCA (**Figure 1A**). However, a negative correlation with immunoregulators was also observed in some cancers, including DLBC, LUAD, LUSC, and TGCT, reflecting the high heterogeneity of MTHFD2 function in various cancers. Moreover, MTHFD2 was discovered to be positively associated with several immune checkpoints, such as cytotoxic T lymphocyte-associated antigen-4 (CTLA-4), PD-L1, T-cell immunoglobulin and mucin domain-3 (TIM-3), and Programmed Cell Death 1 (PD-1) in most cancers, especially in BLCA (**Figures 1B–E**). Then, the immune cells’ infiltration analysis based on the ssGSEA algorithm was performed. The heatmap demonstrated that MTHFD2 expression was correlated with lots of immune cells’ infiltration in cancers. Specifically, MTHFD2 was positively associated with most immune cells in THCA, PAAD, LIHC, KIRP, KIRC, KICH, and BLCA, whereas it was also negatively associated with most immune cells in some other cancers, such as UCEC, SARC, LUSC, and LUAD (**Figure 1F**). Furthermore, we noted that BLCA is one of the cancers with the strongest association between MTHFD2 and TME. Additionally, we noted that MTHFD2 expression was correlated with TMB and MSI in several cancers, indicating its potential value for predicting immunotherapy response in these cancers (**Supplementary Figures 4A, B**). In summary, the expression level of MTHFD2 was associated with immunological features in pan-cancers, including BLCA, and could assess the prognosis of patients.

### MTHFD2 upregulation indicates an inflamed tumor microenvironment in BLCA

**Figure 2:**
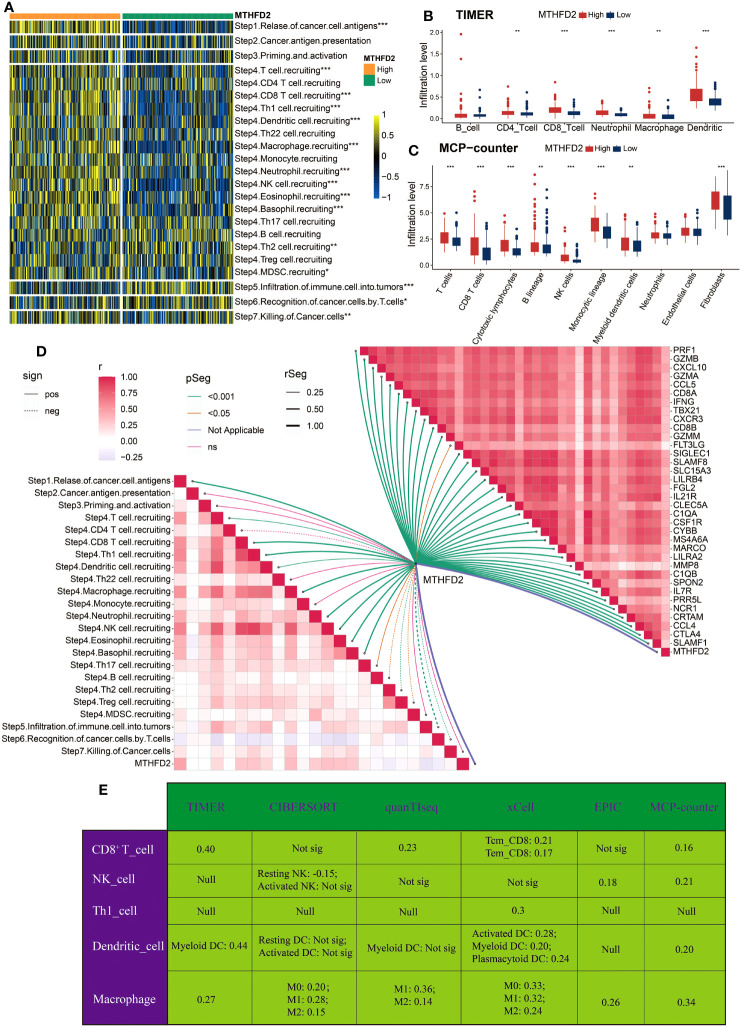
The correlation between MTHFD2 expression and immunological characteristics in the TCGA cohort **(A)** Comparison for enrichment scores of steps involved in cancer immunity cycle between the high- and low-MTHFD2-expression groups in bladder cancer. **(B, C)** Comparison for infiltration level of various immune and stromal cells using TIMER **(B)** and MCP-counter **(C)** algorithms between the high- and low-MTHFD2-expression groups in bladder cancer. **(D)** The correlation analysis between MTHFD2 expression and steps of cancer immunity cycle as well as effector molecules of anti-tumor TIICs (including CD8^+^T cell, macrophages, NK cell, Th1 cell, and dendritic cell). **(E)** The correlation between MTHFD2 expression and infiltration level of various anti-tumor TIICs using six different algorithms (*p< 0.05, **p< 0.01, ***p< 0.001, Not sig refers to not significant, Null means no data for analysis, Tcm refers to central memory T cell, and Tem refers to effector memory T cell).

Considering the association between MTHFD2, immunoregulators, and immune cell infiltrations in pan-cancer, we then investigated its immune-related biological functions in detail in BLCA. The cancer–immunity cycle analysis, which can reflect the stepwise events in anticancer immune response, was performed first. As demonstrated in **Figure 2A**, the activities of most steps were upregulated in the high-MTHFD2 group, indicating an inflamed TME in these patients. The immune cells’ infiltration analyses based on different algorithms, such as TIMER and MCP-counter, were performed as well. As visualized, most immune cells especially T cells and macrophages were highly infiltrated in the high-MTHFD2 group (**Figures 2B, C**). Correlation analysis also revealed a positive correlation between MTHFD2 expression and its effector genes (**Figure 2D**). Moreover, six algorithms were used to further evaluate the correlation between MTHFD2 and anti-tumor TIICs, including CD8^+^ T cells, NK cells, Th1 cells, dendritic cells, and macrophages (**Figure 2E**). These observations indicate that MTHFD2 overexpression could predict an inflamed TME in BLCA patients.

### MTHFD2 could predict the efficacy of immune checkpoint blockade in BLCA

**Figure 3:**
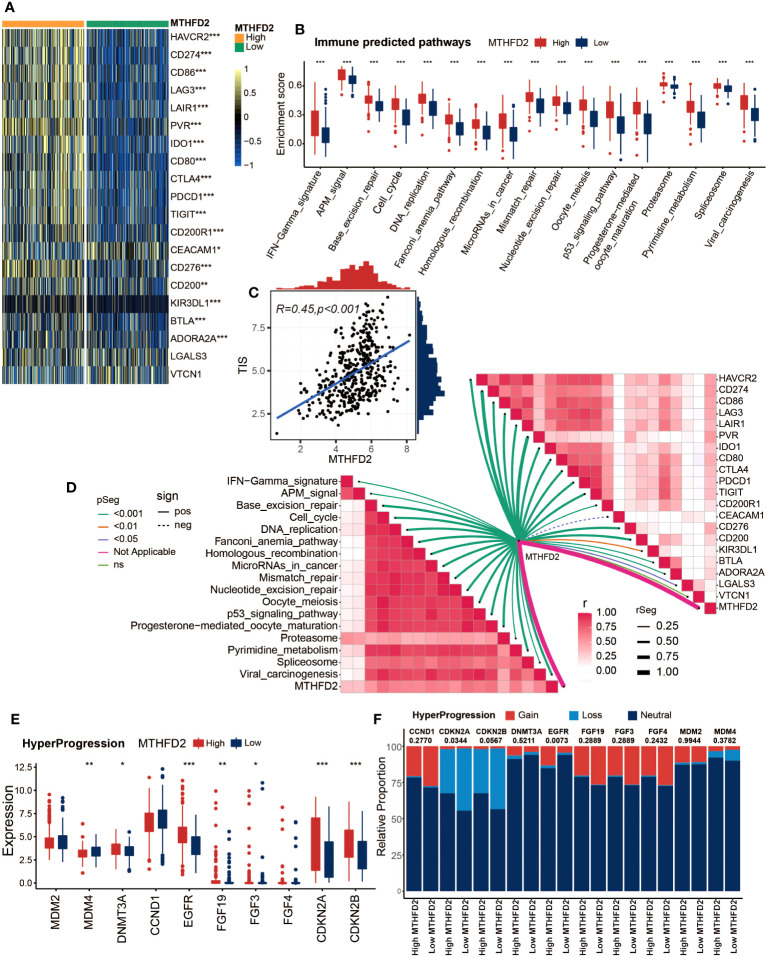
MTHFD2 expression was closely correlated with biomarkers of ICB response in the TCGA cohort. **(A)** Comparison for expression of immune checkpoints between the high- and low-MTHFD2-expression groups in bladder cancer. **(B)** Comparison for enrichment scores of immune predicted pathways between the high- and low-MTHFD2-expression groups in bladder cancer. **(C)** Pearson’s correlation test between MTHFD2 expression and T cell-inflamed scores in bladder cancer in the TCGA cohort. **(D)** The correlation analysis between MTHFD2 expression and enrichment scores of immune predicted pathways as well as expression of various immune checkpoints. **(E, F)** Comparison for expression **(E)** and copy number variation (CNV) **(F)** of hyper-progression-related genes between the high- and low-MTHFD2-expression groups in bladder cancer (*p< 0.05, **p< 0.01, ***p< 0.001, ns means not significant, pos means positive correlation, and neg mean negative correlation).

Given the association between MTHFD2 and the tumor immune microenvironment, we further investigated the potential of MTHFD2 in predicting tumor immunotherapies, such as ICB therapy. Therefore, we explored the relationships between MTHFD2 and several ICB response biomarkers. As shown in **Figure 3A**, most well-known immune checkpoints are significantly enriched in the high-MTHFD2 group, including several clinically widely used ICB targets such as CD274 (PD-L1), PDCD1 (PD-1), and CTLA-4. Moreover, lots of immunotherapy response predicted pathways were also remarkably enriched in the high-MTHFD2 group (**Figure 3B**). The correlation analysis also showed a positive correlation between MTHFD2 expression and immune inhibitory genes (**Figure 3D**). The TIS, which could predict the efficacy of ICB, was evaluated as well, and a significant positive correlation between TIS and MTHFD2 was discovered via Pearson’s correlation test (**Figure 3C**). These results suggested that patients with high MTHFD2 expression are apt to respond to immunotherapies. Furthermore, considering the significant adverse effect of hyperprogression on patients’ prognosis, we also investigated the relationships between MTHFD2 and several hyperprogression predictors. CDKN2A and CDKN2B, whose amplification are adversely associated with the possibility of hyperprogression, were observed to be significantly elevated in the high-MTHFD2 group and showed a lower frequency of copy number loss, suggesting a lower possibility of hyperprogression in these patients (**Figures 3E, F**). However, the correlations of MTHFD2 with genes positively associated with the possibility of hyperprogression varied. While some genes were significantly downregulated in the high-MTHFD2 group or showed a similar tendency, such as MDM4 and CCND1, some other genes were upregulated, such as DNMT3A, EGFR, FGF19, and FGF3 (**Figures 3E, F**). Thus, further investigations are needed.

### MTHFD2 expression could precisely predict the molecular subtypes and possible therapeutic strategies in BLCA

**Figure 4:**
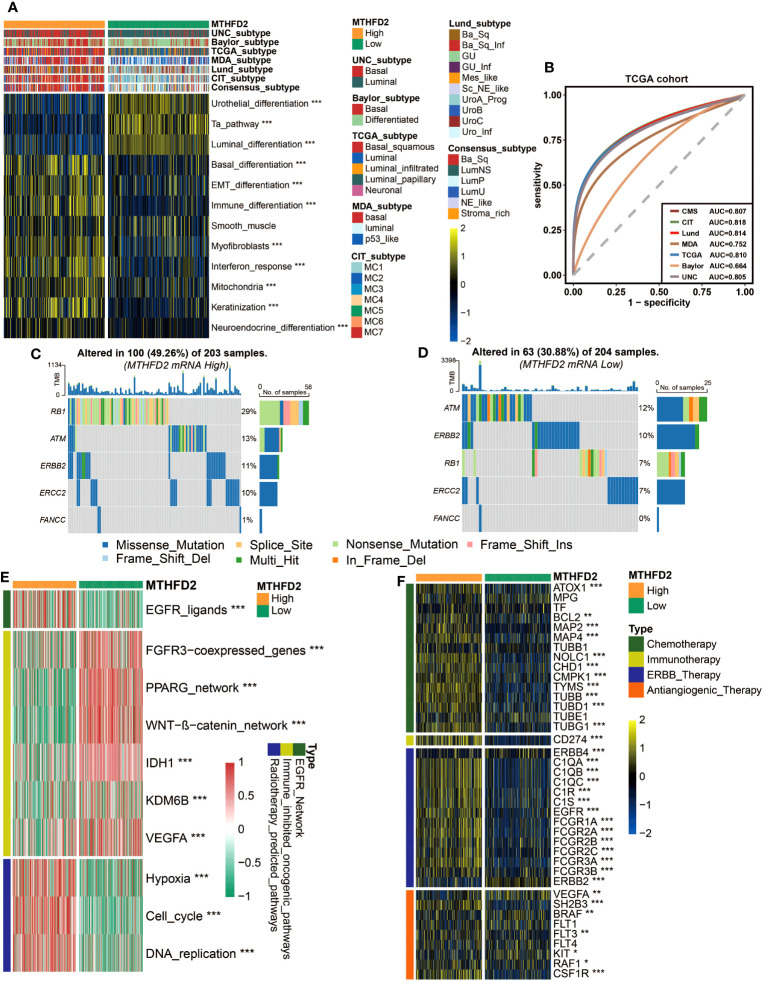
MTHFD2 expression was closely related to the molecular subtype and predictive biomarkers of therapeutic effects of various clinical treatment strategies of BLCA in TCGA cohort. **(A)** Distribution of bladder cancer molecular subtypes calculated via multiple algorithms in patients with different MTHFD2 expression levels. **(B)** Receiver operating characteristics (ROC) curve and area under the curve (AUC) for MTHFD2 in molecular subtype prediction in the TCGA cohort. **(C, D)** The mutation profile of neoadjuvant-related molecules in the high- and low-MTHFD2-expression groups. **(E)** Comparison for enrichment scores of different therapeutic strategies in the high- and low-MTHFD2-expression groups in bladder cancer. **(F)** Comparison for expression of various drug-targeted genes in the high- and low-MTHFD2-expression groups in bladder cancer (*p< 0.05, **p< 0.01, ***p< 0.001).

Owing to the heterogeneity of BLCA and the tight associations between molecular subtypes and patients’ prognosis and treatment outcomes, the molecular subtypes of patients with different MTHFD2 expression levels were analyzed based on seven classification systems. As shown in **Figure 4A**, basal-like subtypes, which are considered to be more aggressive but also apt to respond to certain therapies such as immunotherapy and anti-EGFR therapy, were found to be enriched in the high-MTHFD2 group. This finding was further confirmed by the enrichment analysis of molecular subtype-related pathways. The high-MTHFD2 group showed significantly increased activity in pathways including basal differentiation, EMT differentiation, immune differentiation, myofibroblasts, interferon response, mitochondria, keratinization, and neuroendocrine differentiation. In contrast, the low-MTHFD2 group exhibited higher activity in pathways including urothelial differentiation, Ta pathway, and luminal differentiation (**Figure 4A**). Furthermore, apart from MDA and Baylor systems, the value of area under curve (AUC) of all other subtyping systems was larger than 0.8, suggesting the high accuracy of MTHFD2 in predicting molecular subtypes (**Figure 4B**). Similar results were validated in two independent cohorts (E-MTAB-432 and IMvigor210 cohorts), further increasing the reliability of the conclusions (**Supplementary Figures 4C, D**).

The association between MTHFD2 and molecular subtypes of BLCA suggested that except for the previously mentioned immunotherapies, MTHFD2 could have the potential to predict the efficacy of other therapies as well. Thus, we explored the relationship between MTHFD2 expression and many therapeutic effect-related biomarkers, including the mutation profile of neoadjuvant-related molecules, several therapeutic signatures, and targeted drug-related gene expression. The high-MTHFD2 group showed a higher mutation frequency of several neoadjuvant chemotherapy-related genes, especially RB1 and ERCC2 (**Figures 4C, D**). Moreover, the EGFR ligands and radiotherapy-predicted pathways were enriched in the high-MTHFD2 group, while the immune inhibited oncogenic pathways, whose activation was associated with non-inflamed TME and immunotherapy resistance (53–55), were enriched in the low-MTHFD2 group (**Figure 4E**). Furthermore, the expression of lots of drug-targeted genes was observed to be significantly higher in the high-MTHFD2 group (**Figure 4F**). Specifically, almost all targets of chemotherapy, immunotherapy, and ERBB (epidermal growth factor) therapy, as well as some targets of anti-angiogenic therapy, such as SH2B3, RAF1, and CSF1R, were elevated in the high-MTHFD2 group. However, there were also some targets of anti-angiogenic therapy found to be underexpressed in the high-MTHFD2 group (**Figure 4F**). These findings suggested that neoadjuvant and adjuvant chemotherapy, radiotherapy, immunotherapy, and ERBB-family-based target therapy could be ideal choices for BLCA patients with high MTHFD2 expression.

### Validating the role of MTHFD2 in the GSE48075 cohort

**Figure 5:**
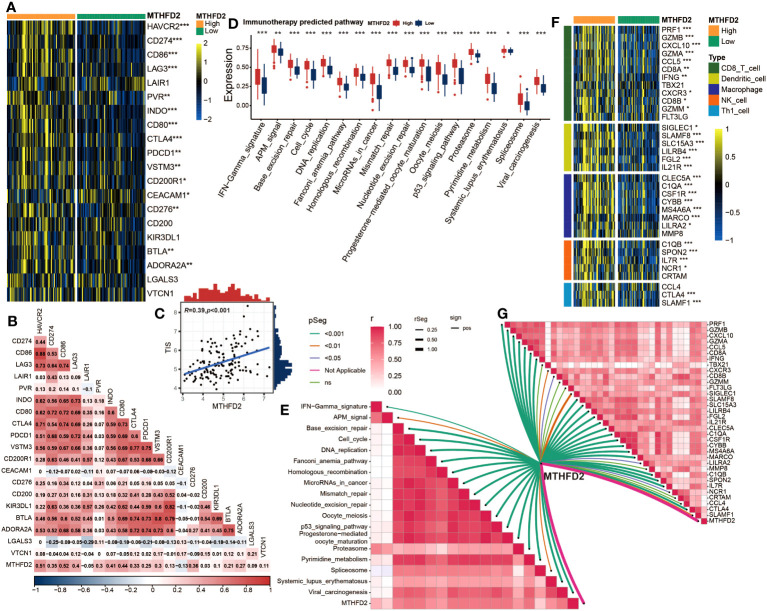
Validation of the correlation between MTHFD2 expression and biomarkers of ICB response in the GSE48075 cohort **(A)** Comparison for expression of immune checkpoints between the high- and low-MTHFD2-expression groups in bladder cancer. **(B)** The correlation between MTHFD2 expression and immune checkpoints using Pearson’s correlation analysis. **(C)** Correlation between MTHFD2 expression and T cell-inflamed scores in bladder cancer in the GSE48075 cohort. **(D)** Comparison for enrichment scores of immune predicted pathways between the high- and low-MTHFD2-expression groups in bladder cancer. **(E)** The correlation analysis between MTHFD2 expression and enrichment scores of immune predicted pathways. **(F)** Comparison for expression of effector genes derived from anti-tumor cells in the high- and low-MTHFD2-expression groups. **(G)** The correlation analysis between MTHFD2 expression and expression of effector genes derived from anti-tumor cells (*p< 0.05, **p< 0.01, ***p< 0.001, ns means not significant, pos means positive correlation, and neg mean negative correlation).

Next, considering the possible biases of bioinformatic analysis, we validated the findings mentioned above in an independent external cohort (GSE48075 cohort). MTHFD2 was validated to be positively correlated with several immune checkpoints, including PDCD1 (PD-1), CD274 (PD-L1), and CTLA-4. Higher MTHFD2 expression tend to be accompanied by higher expression of these immune checkpoints (**Figures 5A, B**). Furthermore, the positive correlation between MTHFD2 expression and TIS was also confirmed via Pearson’s correlation test (**Figure 5C**). Furthermore, the enrichment scores of all immunotherapy predicted pathways and the expression of effector genes for most anti-tumor immune cells were elevated in patients with high MTHFD2 expression (**Figures 5D–G**).

**Figure 6:**
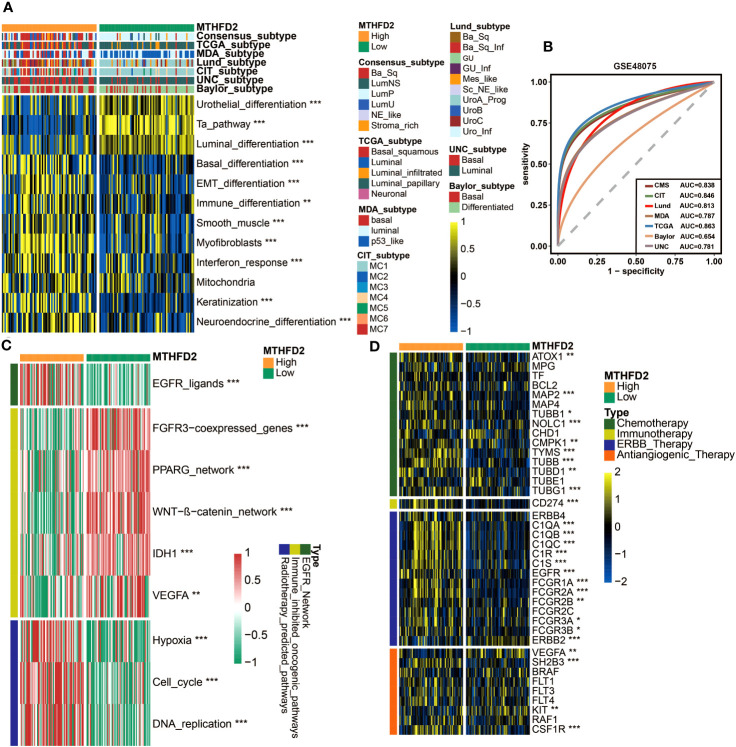
MTHFD2 expression was closely related to the molecular subtype and response for various clinical treatment strategies of bladder cancer in the GSE48075 cohort. **(A)** Distribution of bladder cancer molecular subtypes calculated via multiple algorithms in patients with different MTHFD2 expression levels. **(B)** Receiver operating characteristics (ROC) curve and area under curve (AUC) for MTHFD2 in molecular subtype prediction in the GSE48075 cohort. **(C)** Comparison for enrichment scores of various therapeutic effects predictive signature in the high- and low-MTHFD2-expression groups in bladder cancer. **(D)** Comparison for expression of various drug-targeted genes in the high- and low-MTHFD2-expression groups in bladder cancer (*p< 0.05, **p< 0.01, ***p< 0.001).

In addition, MTHFD2 was validated to have the potential to accurately distinguish the molecular subtypes of BLCA in the GSE48075 cohort, in terms of the basal and luminal subtypes (**Figures 6A, B**). As for the relationships with therapeutic biomarkers, the results of GSE48075 were similar with those from the TCGA-BLCA cohort, confirming the predictive value of MTHFD2 in the efficacy of chemotherapy, radiotherapy, immunotherapy, and targeted therapy (**Figures 6C, D**).

Collectively, the association of MTHFD2 expression with TME, molecular subtypes, and efficacy of multiple treatments was validated in an independent external cohort.

### Validating the role of MTHFD2 in distinguishing immunophenotype and the clinical response of ICB by immunohistochemistry and the IMvigor210 cohort

**Figure 7:**
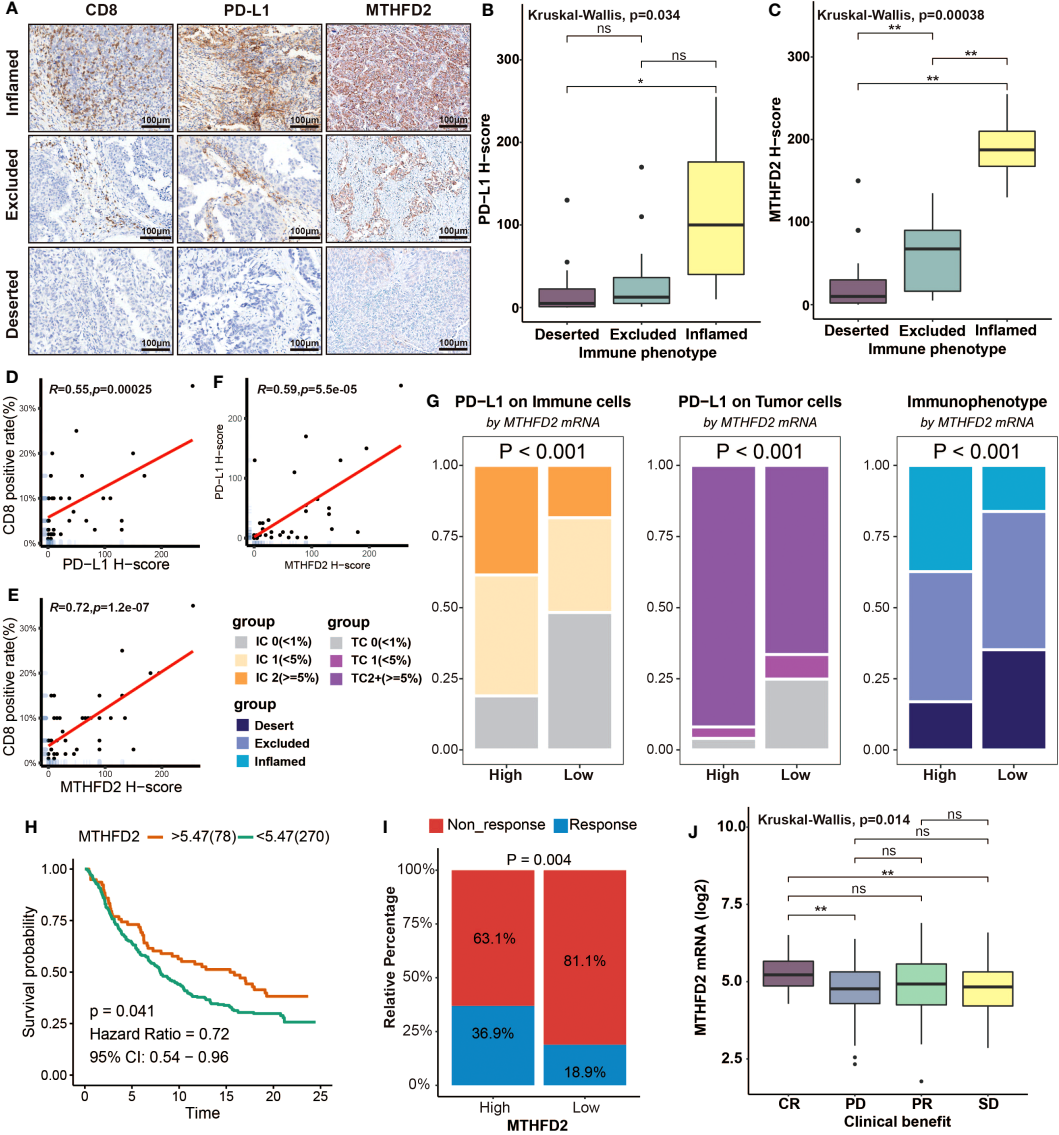
Overexpression of MTHFD2 represents an inflamed tumor microenvironment, indicating the favorable response of immunotherapy. **(A)** The immunochemistry staining of CD8, PD-L1, and MTHFD2 on BLCA samples with different immunophenotypes. **(B, C)** Comparison for the H-score of PD-L1 and MTHFD2 in different immune phenotypes based on immunohistochemistry. **(D)** The correlation between the CD8-positive rate and H-score of PD-L1. **(E)** The correlation between the CD8-positive rate and H-score of MTHFD2. **(F)** The correlation between the H-score of PD-L1 and MTHFD2. **(G)** The proportions of immune (left) and tumor (middle) cells with different PD-L1 expression levels and the fraction of various immunophenotypes (right) in the high- and low-MTHFD2-expression groups based on the IMvigor210 cohort. **(H)** Survival curves of patients receiving immunotherapy (Atezolizumab/anti-PD-L1) treatment based on MTHFD2 expression. **(I)** Comparison for the response rate of immunotherapy (Atezolizumab/anti-PD-L1) treatment between the high- and low-MTHFD2-expression groups. **(J)** The different expression level of MTHFD2 between people with different clinical benefit (*p< 0.05, **p< 0.01, ns refers to not significant, TC refers to tumor cells, IC refers to immune cells).

To further investigate the relationship between MTHFD2 and TME, we performed immunohistochemistry staining on BLCA samples. These samples were divided into three immunophenotypes (inflamed, excluded, and deserted) according to a previous study (63), based on the infiltration of CD8^+^ T cells into tumors. The samples of the inflamed type also exhibited the highest PD-L1 expression. As for MTHFD2, it was found to be most abundant in the inflamed phenotype, but significantly lower in excluded phenotypes and absent in deserted phenotypes (**Figure 7A**). Boxplot showed the evaluated H-score of PD-L1 and MTHFD2 in three different immune phenotypes. The H-scores of PD-L1 and MTHFD2 were significantly lower in the deserted group than in the inflamed group. However, unlike the H-score of MTHFD2, the H-score of PD-L1 was observed with no significant difference between the inflamed group and the excluded group (**Figures 7B, C**). Furthermore, significant positive relationships were observed between the positive rate of CD8^+^ T cells, the H-score of PD-L1, and MTHFD2 in correlation analysis (**Figures 7D–F**). These findings were further validated in the IMvigor210 cohort. The high-MTHFD2 group had significantly higher proportion of IC 2^+^ (immune cells showing highest PD-L1 level), TC 2^+^ (tumor cells showing highest PD-L1 level), and the inflamed phenotype (**Figure 7G**). Moreover, the prognosis and response rate of anti-PD-L1 therapy were significantly better in the high-MTHFD2 group. The patients with better clinical benefit also showed higher MTHFD2 expression, indicating the predictive value of MTHFD2 in immune response of immunotherapy (**Figures 7H–J**).

In conclusion, overexpression of MTHFD2 was closely related to an inflamed TME and showed robust predictive value in predicting immunotherapy response.

## Discussion

MTHFD2, a critical enzyme of folate metabolism, was found to be associated with various malignant features and patients’ prognosis in several cancers (19–24). Recently, some studies revealed that it was also involved in the regulation of immune cells (25, 26). However, its detailed roles in cancer immunity and its relationship to cancer immunotherapy remain obscure. In this study, we briefly assessed the prognostic value and immunological function of MTHFD2 in pan-carcinoma, and further investigated its relationship with TME, molecular subtypes, and immunotherapy response in BLCA in detail.

The pan-cancer co-expression analysis revealed that MTHFD2 was positively correlated with most immunoregulators, including immune checkpoints, in most cancers. These findings confirmed the wide involvement of MTHFD2 in cancer immunity and its association with some immune checkpoints (26). Furthermore, MTHFD2 was observed to be positively correlated with infiltrations of several immune cells in various cancers, especially the activated CD4^+^ T cell and type 2 T helper cell, which were significant in almost all cancers, suggesting that MTHFD2 might be related to the inflamed TME. This result is in line with a prior study demonstrating that MTHFD2 plays a critical role in maintaining the *de novo* purine synthesis of activated CD4^+^ T cells (25). Notably, THCA, LIHC, KIRP, KIRC, KICH, and BLCA reached significance in almost all co-expression analysis and immune cell infiltration analysis, indicating that these cancers may be a suitable candidate for MTHFD2-based therapy or predictive model. In prognostic value analysis, we discovered that high MTHFD2 expression was a risky factor for patients’ survival in many cancer types, which is in line with previous studies and the theory that high MTHFD2 expression can help cancer cells meet the high biosynthetic requirement (19–24).

Then, in detail, we explored the immunological functions of MTHFD2 in BLCA. The cancer–immunity cycle analysis, which can reflect the immune system regulation step by step, revealed that high MTHFD2 was correlated with higher score of most steps in immune response, including T-cell recruiting, NK cell recruiting, and killing of cancer cells. Consistently, the infiltration of several typical anti-tumor TIICs, including CD8^+^ T cell (64), NK cell (65), type 1 T helper cell (66), dendritic cell (67), and macrophage (68), was significantly higher in patients with high MTHFD2 expression. The expression of effector genes of these anti-tumor TIICs was also positively related with MTHFD2 expression. Moreover, similar results were observed in validation analysis performed on the external cohort GSE48075. Combining with these findings, we conclude that the high expression of MTHFD2 can predict an inflamed TME in BLCA.

As previous studies illustrated, tumors with an inflamed TME have a higher possibility to respond to immunotherapy, including ICB therapy (69–71). Thus, we subsequently assessed the association between MTHFD2 and immunotherapy efficacy. The results illustrated that MTHFD2 was positively correlated with most immune checkpoints and immunotherapy response predictive pathways, as well as the TIS, which can predict the effectiveness of ICB (64). These findings were validated in the GSE48075 cohort as well. Furthermore, the MTHFD2 expression was found to be related to the expression level and copy number variance of several hyperprogression predictive genes, suggesting its potential to predict the hyperprogression in BLCA patients treated with ICB (63). Collectively, our results illustrated that the high expression of MTHFD2 may be a predictor of good outcomes of immunotherapy in BLCA patients.

Considering the predictive value of molecular subtype for patients’ prognosis and treatment responses, we then evaluated the association of MTHFD2 with molecular subtypes based on different classification systems. Patients with high MTHFD2 expression showed significantly higher proportion of the basal-like subtype, which are considered to be more aggressive but also respond better to some treatments, such as chemotherapy, ICB therapy, and EGFR-based targeted therapy (14, 31, 58–60, 62, 72). The basal-type associated pathways were enriched in the high-MTHFD2 group as well. Additionally, the AUC value of receiver operating characteristics (ROCs) suggested that the accuracy of MTHFD2 in predicting molecular subtypes is pretty high, further enhancing its potential value in clinical practice. The relationships of MTHFD2 with other biomarkers that can predict the therapeutic effects were analyzed too. Consistent with molecular subtype analysis, the high-MTHFD2 group showed higher mutation frequency of chemotherapy response predictive genes, higher enrichment of radiotherapy predictive pathways and EGFR ligands, lower enrichment of immunotherapy resistance predictive pathways, and higher expression of many drug-targeted genes. These findings suggested that MTHFD2 can not only predict the immunotherapy efficacy, but also have the potential to predict the efficacy of many other anti-cancer treatments, increasing its value in clinical practice again. In addition, similar results in the validation group also enhance the certainty of these conclusions.

Finally, we verified the association of MTHFD2 with TME and immunotherapy efficacy by immunohistochemistry and RNA-seq analysis in the IMvigor210 cohort. According to the IHC assay and analyses results of the IMvigor210 cohort, higher MTHFD2 expression was found to be related with higher proportion of inflamed immune phenotype, which indicates a high infiltration degree of TIICs and a higher rate of ICB response (73). Consistently, the PD-L1 expression was elevated in patients with high MTHFD2 expression as well. Furthermore, the clinical data of the IMvigor210 cohort confirmed the correlation between MTHFD2 and ICB therapy efficacy directly. Patients in the high-MTHFD2 group showed a higher proportion to respond to anti-PD-L1 therapy and benefited more from it. It was noteworthy that, in the IMvigor210 cohort, the survival length of the high-MTHFD2 group was also remarkably longer than that of the low-MTHFD2 group, as this was contrary to the prognosis data of the TCGA database, which correlated high MTHFD2 with shorter OS and PFS. Considering that all patients of the IMvigor210 cohort received atezolizumab therapy (1,200 mg, every 3 weeks), we thought that this was due to the significant survival benefits from ICB therapy in patients with high MTHFD2 expression.

Collectively, different from previous studies that mainly focused on its roles in cancer cells, such as promoting cancer cell proliferation and participating in transcriptional regulation and metabolic reprogramming, regarding MTHFD2 as a novel promising target for cancer therapy (23, 24, 74), our study concentrated on its correlation with cancer immunity and revealed that MTHFD2 could be a robust biomarker for immunotherapy outcomes in BLCA. Given that the inhibitors of MTHFD2 are still under development, immunotherapy might be an alternative option for patients with high MTHFD2 expression.

However, there are several limitations in our study as well. First of all, although external cohorts were adopted to make validation, our conclusion is still largely based on bioinformatic analysis of data from public databases, with limited exploration for specific mechanisms. Therefore, further investigation focusing on the detailed processes of MTHFD2 in regulating cancer immunity is needed in the future. Secondly, the patient samples from Shanghai Outdo Biotech Company lacked corresponding follow-up information and immunotherapy efficacy data; hence, the efficacy data of ICB therapy in the IMvigor210 cohort, which could be influenced by random effects and biases, have not been independently verified. Thus, further clinical research is clearly necessary. Lastly, although we have verified the conclusions of the public databases by immunohistochemistry to some extent, more experiments are still needed to reduce the inevitable biases brought by bioinformatics analysis.

## Data availability statement

The datasets presented in this study can be found in online repositories. The names of the repository/repositories and accession number(s) can be found in the article/**Supplementary Material**.

## Ethics statement

The studies involving humans tissue samples were approved by Ethics Committee of Shanghai Outdo Biotech Company. The human samples used in this study were acquired from Shanghai Outdo Biotech Company. The studies were conducted in accordance with the local legislation and institutional requirements.

## Author contributions

XS: Data curation, Formal Analysis, Funding acquisition, Investigation, Validation, Visualization, Writing – original draft. XP: Data curation, Investigation, Software, Validation, Writing – review & editing. YC: Data curation, Formal Analysis, Investigation, Writing – review & editing. ZS: Formal Analysis, Investigation, Visualization, Writing – review & editing. CY: Validation, Visualization, Writing – review & editing. LZ: Conceptualization, Funding acquisition, Project administration, Writing – review & editing. LFZ: Conceptualization, Funding acquisition, Project administration, Writing – review & editing. SG: Conceptualization, Funding acquisition, Project administration, Supervision, Writing – review & editing.
