# Supplementary material for: Overexpression of MTHFD2 represents an inflamed tumor microenvironment and precisely predicts the molecular subtype and immunotherapy response of bladder cancer
Source: Front Immunol. 2023 Dec 7;14:1326509. doi: 10.3389/fimmu.2023.1326509 (PMC10733511; doi:10.3389/fimmu.2023.1326509)

Supplementary Material

Supplementary Figure 1. The prognostic value of MTHFD2 in pan-carcinoma in terms of overall survival (OS). (A) Forest map of univariate cox analysis for MTHFD2 in cancers in terms of OS. (B-I) Kaplan-Meier survival curve for MTHFD2 in various cancer types in terms of OS.

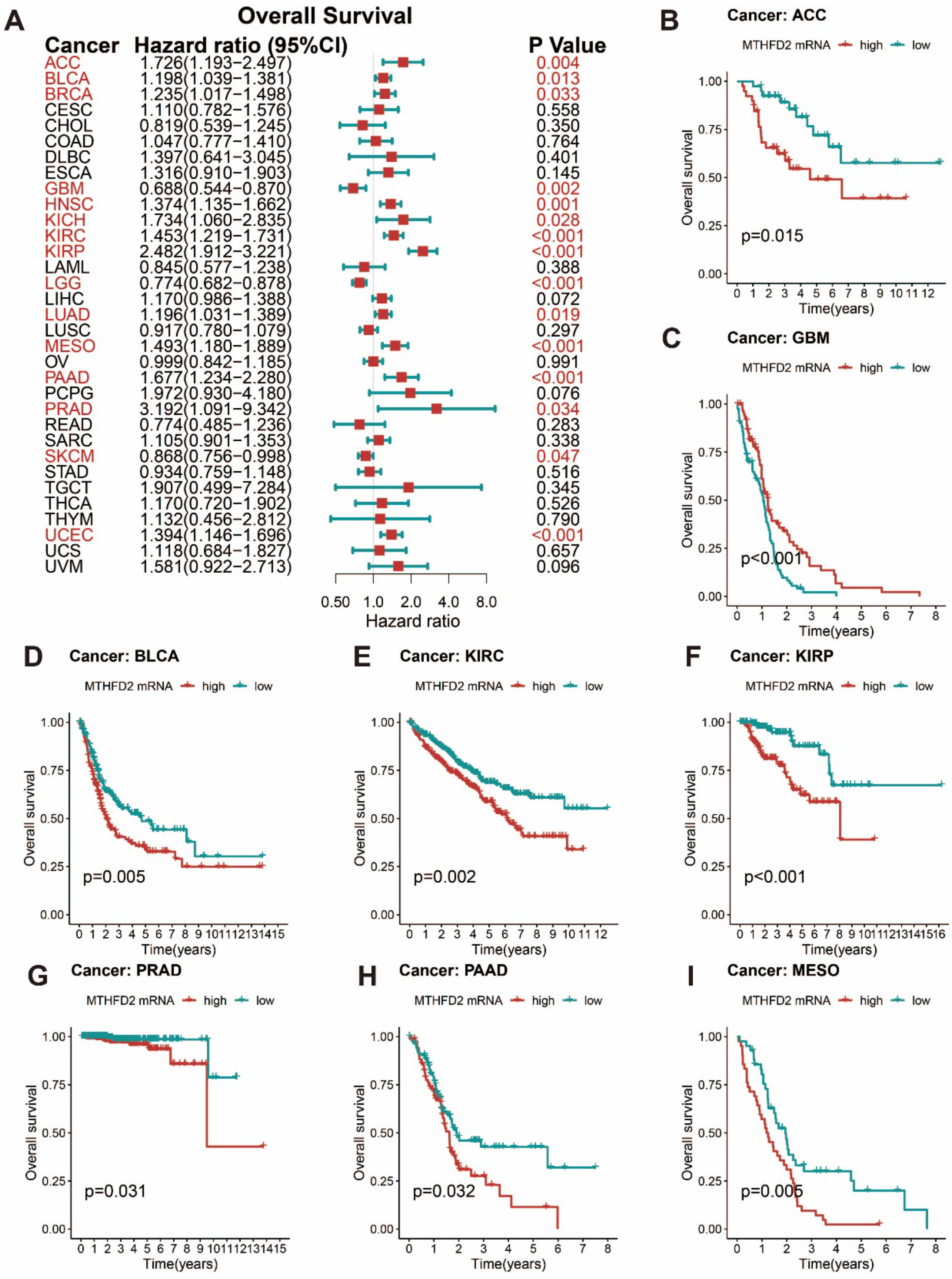

Supplementary Figure 2. The prognostic value of MTHFD2 in pan-cancers in terms of progression-free survival (PFS). (A) Forest map of univariate cox analysis for MTHFD2 in pan-cancers in terms of PFS. (B-I) Kaplan-Meier survival curve for MTHFD2 in various cancer types in terms of PFS.

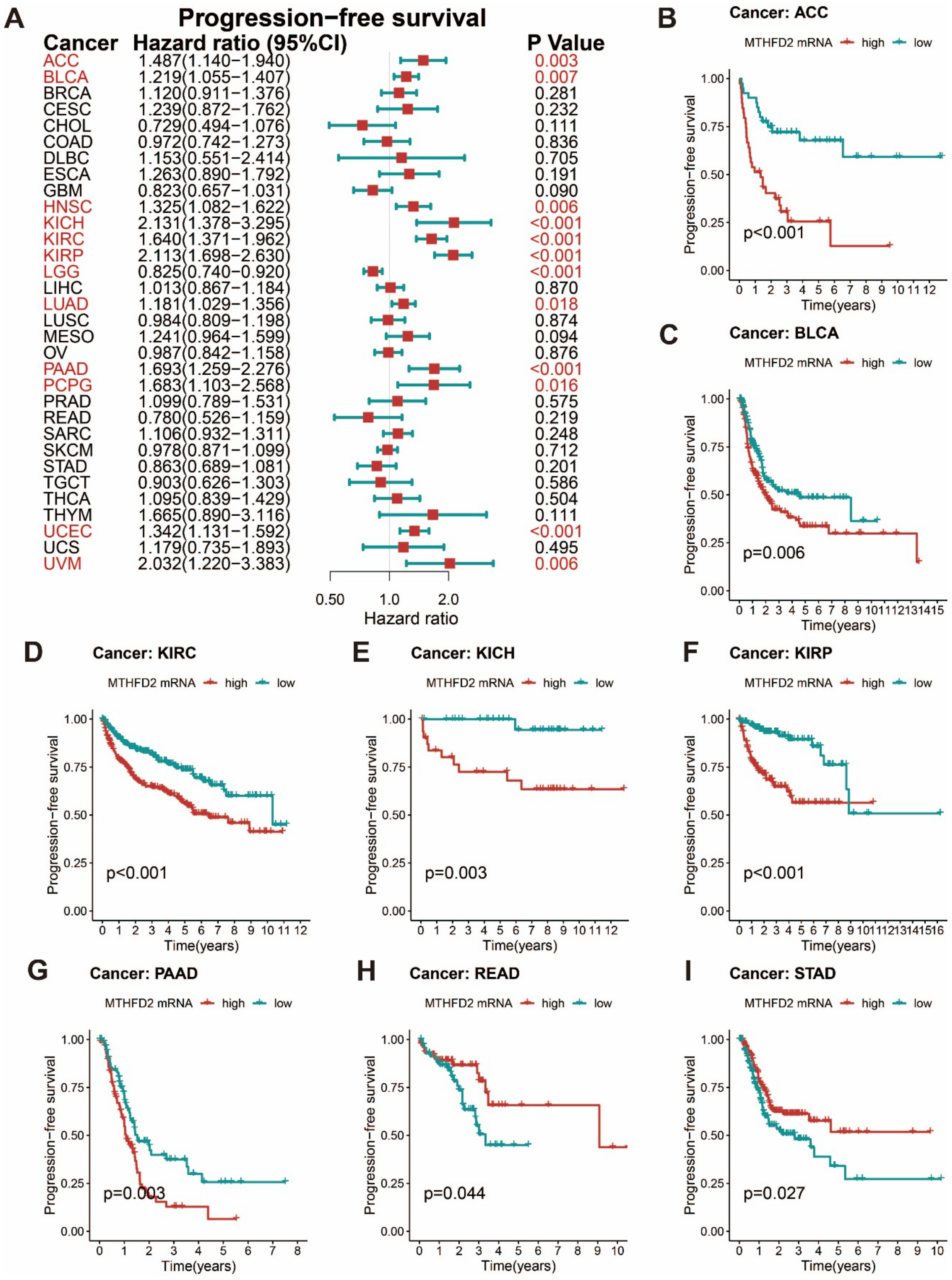

Supplementary Figure 3. The prognostic value of MTHFD2 in pan-cancers in terms of Disease-free survival (DFS). (A) Forest map of univariate cox analysis for MTHFD2 in pan-cancers in terms of DFS. (B-I) Kaplan-Meier survival curve for MTHFD2 in various cancer types in terms of DFS.

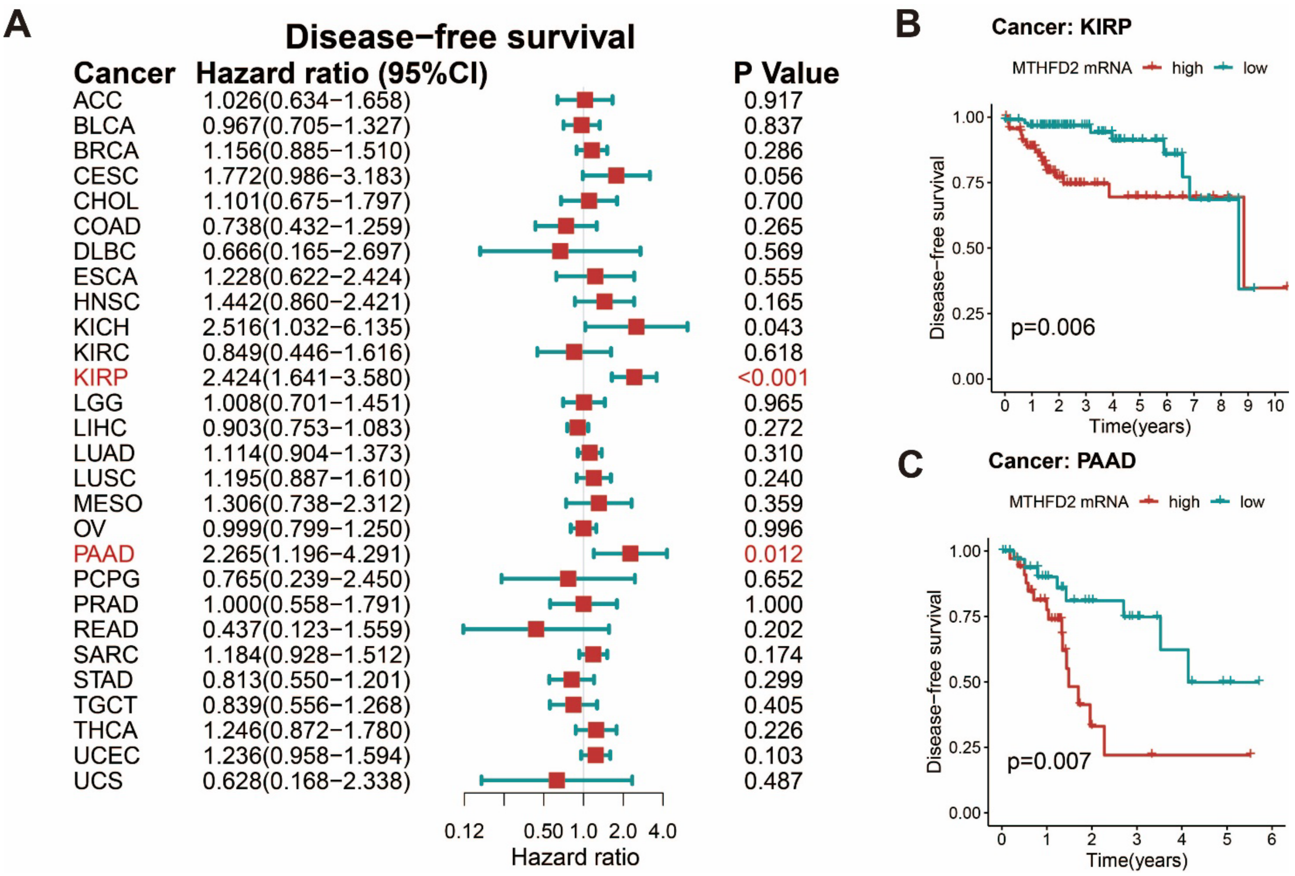

Supplementary Figure 4. The correlation between MTHFD2 expression and tumor mutation burden (TMB), Microsatellite Instability (MSI), Molecular subtype. (A) The correlation between MTHFD2 and TMB based on Pearson's coefficient. (B) The correlation between MTHFD2 and MSI status based on Spearman coefficient. (C-D) Receiver operating characteristics (ROC) curve for MTHFD2 in molecular subtype prediction with corresponding area under curve (AUC) in E-MTAB-4321 and IMvigor210 cohort, respectively. (*P<0.05, ** P <0.01, *** P <0.001)

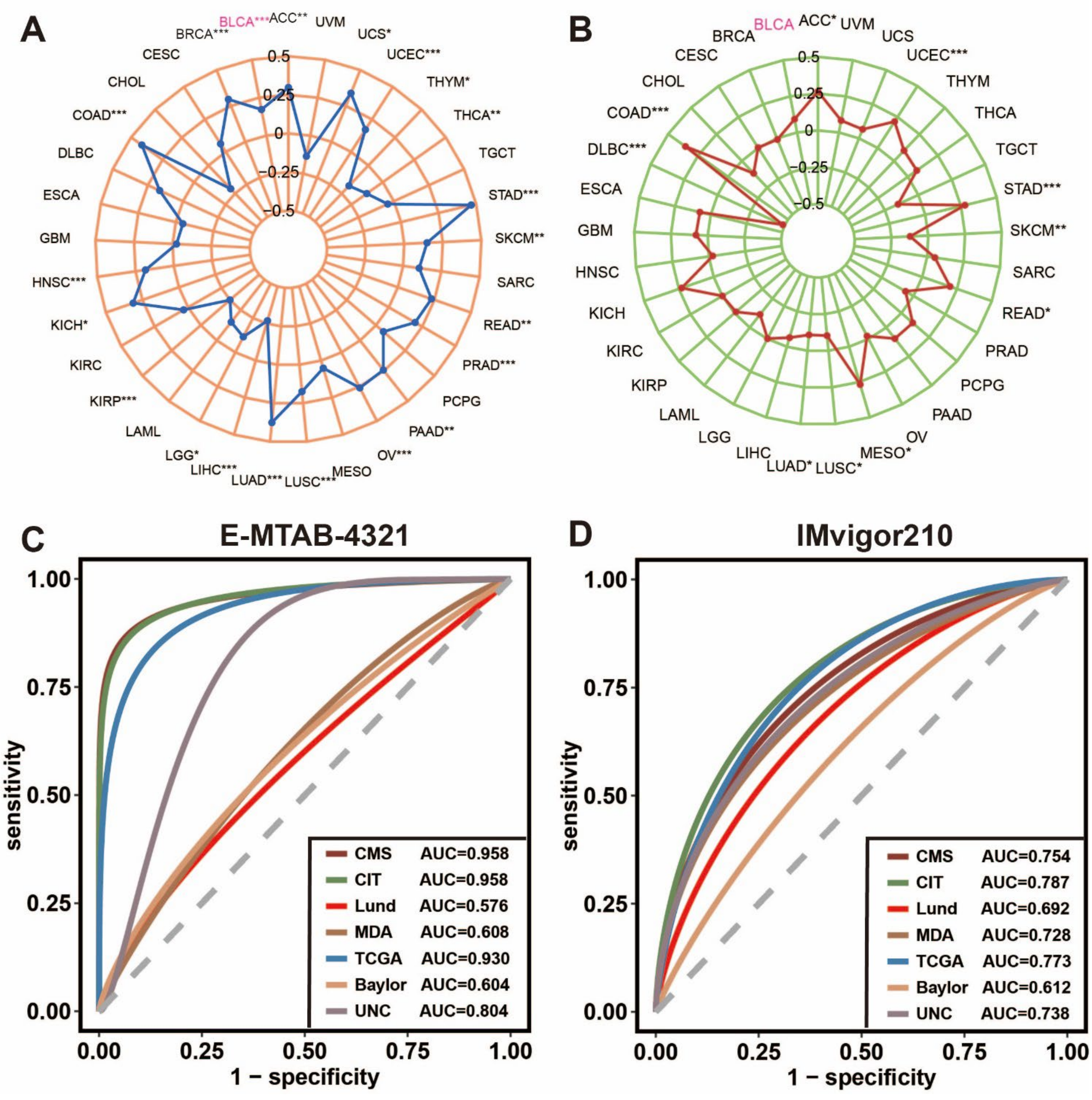

Supplement: Supplementary file 1 [file DataSheet_1.pdf]
